# End-of-life decisions in Greek intensive care units: a multicenter cohort study

**DOI:** 10.1186/cc9380

**Published:** 2010-12-20

**Authors:** Georgios Kranidiotis, Vasiliki Gerovasili, Athanasios Tasoulis, Elli Tripodaki, Ioannis Vasileiadis, Eleni Magira, Vasiliki Markaki, Christina Routsi, Athanasios Prekates, Theodoros Kyprianou, Phyllis-Maria Clouva-Molyvdas, Georgios Georgiadis, Ioannis Floros, Andreas Karabinis, Serafim Nanas

**Affiliations:** 1 First Critical Care Department, Evangelismos Hospital, National and Kapodistrian University of Athens, 45-47 Ypsilantou Str, Athens, 10675, Greece; 2 Department of Clinical Therapeutics, Alexandra Hospital, National and Kapodistrian University of Athens, 80 Vasilissis Sofias Av, Athens, 11528, Greece; 3 Critical Care Department, Thriassio General Hospital, G. Gennimata Av, Eleusis, 19600, Greece; 4 Critical Care Department, Tzaneio General Hospital, Afentouli & Zanni Str., Piraeus, 18536, Greece; 5 Critical Care Department, Nicosia General Hospital, 215 Old Road Nikosia-Limassol, Nikosia, 2029, Cyprus; 6 Critical Care Department, Metropolitan Hospital, Ethnarhou Makariou & 1 Eleutheriou Venizelou Str., Athens, 18547, Greece; 7 Critical Care Department, Laiko General Hospital, 17 Aghiou Thoma Str., Athens, 11527, Greece; 8 Critical Care Department, G. Gennimatas General Hospital, 154 Mesogeion Av, Athens, 11527, Greece

## Abstract

**Introduction:**

Intensive care may prolong the dying process in patients who have been unresponsive to the treatment already provided. Limitation of life-sustaining therapy, by either withholding or withdrawing support, is an ethically acceptable and common worldwide practice. The purpose of the present study was to examine the frequency, types, and rationale of limiting life support in Greek intensive care units (ICUs), the clinical and demographic parameters associated with it, and the participation of relatives in decision making.

**Methods:**

This was a prospective observational study conducted in eight Greek multidisciplinary ICUs. We studied all consecutive ICU patients who died, excluding those who stayed in the ICU less than 48 hours or were brain dead.

**Results:**

Three hundred six patients composed the study population, with a mean age of 64 years and a mean APACHE II score on admission of 21. Of study patients, 41% received full support, including unsuccessful cardiopulmonary resuscitation (CPR); 48% died after withholding of CPR; 8%, after withholding of other treatment modalities besides CPR; and 3%, after withdrawal of treatment. Patients in whom therapy was limited had a longer ICU (*P *< 0.01) and hospital (*P *= 0.01) length of stay, a lower Glasgow Coma Scale score (GCS) on admission (*P *< 0.01), a higher APACHE II score 24 hours before death (*P *< 0.01), and were more likely to be admitted with a neurologic diagnosis (*P *< 0.01). Patients who received full support were more likely to be admitted with either a cardiovascular (*P *= 0.02) or trauma diagnosis (*P *= 0.05) and to be surgical rather than medical (*P *= 0.05). The main factors that influenced the physician's decision were, when providing full support, reversibility of illness and prognostic uncertainty, whereas, when limiting therapy, unresponsiveness to treatment already offered, prognosis of underlying chronic disease, and prognosis of acute disorder. Relatives' participation in decision making occurred in 20% of cases and was more frequent when a decision to provide full support was made (*P *< 0.01). Advance directives were rare (1%).

**Conclusions:**

Limitation of life-sustaining treatment is a common phenomenon in the Greek ICUs studied. However, in a large majority of cases, it is equivalent to the withholding of CPR alone. Withholding of other therapies besides CPR and withdrawal of support are infrequent. Medical paternalism predominates in decision making.

## Introduction

Intensive care may prolong the dying process in patients who have been unresponsive to the treatment already provided and for whom the possibility of surviving or regaining an acceptable quality of life is nil. Withholding and withdrawal of life-sustaining treatment were introduced to avoid the futile suffering of dying patients. These practices are based on the principles of bioethics; they are common worldwide, have been approved by the international scientific community, and must not be confused with euthanasia [1,2].

Observational studies conducted in several countries on different continents showed that a large proportion of intensive care unit (ICU) deaths are preceded by withholding or withdrawal of treatment, and that a variety of clinical parameters are associated with the decision to limit treatment [3-12]. The frequency of withholding or withdrawal of treatment and the degree of involvement of relatives in the decision making are influenced by the cultural context [13,14].

The objective of this multicenter study was to study the frequency, types, and rationale for limiting life support in Greek multidisciplinary ICUs, the clinical and demographic parameters associated with it, and the participation of relatives in the decision-making process.

## Materials and methods

This was a prospective observational study conducted in eight multidisciplinary, general hospital-affiliated ICUs (seven in Athens, and one in Nicosia, Cyprus). The contribution of each ICU and the dates defining the periods of data collection are presented in Table 1. In terms of the number of beds, the participating ICUs represent about one third of the total in Greece and Cyprus. We studied all consecutive ICU patients who died, excluding those who stayed in the ICU less than 48 hours or were diagnosed with brain death.

**Table 1:** Periods of data collection and contributions of individual ICUs

ICU	Period of data collection	Patients admitted	Patients included in the study
1	27/11/06 to 26/11/07 and 1/10/08 to 31/5/09	763	159
2	1/1/08 to 15/11/08	137	28
3	15/9/08 to 10/12/08	66	14
4	10/9/08 to 31/5/09	312	26
5	19/1/09 to 22/9/09	115	25
6	1/10/08 to 31/8/09	92	15
7	1/8/09 to 31/10/09	114	8
8	1/3/09 to 31/8/09	441	31

Total		2,040	306

ICU, intensive care unit.

### The physician in charge of each study patient was invited

1. To classify the patient into one of four mutually exclusive categories: patients who received full support, including unsuccessful cardiopulmonary resuscitation (CPR) (group A); those who received active support up to but not including CPR (group B); those with a decision to withhold (not to start/escalate) some form of life support besides CPR (group C); or those with a decision to withdraw an existing form of life support (group D).

2. To complete an anonymous questionnaire, indicating the factors that influenced his or her decision to offer full support or to limit therapy (choosing them from among a list of prespecified items and weighing them on a scale ranging from 0 for no impact to 4 for ultimate impact), the degree and nature of relatives' involvement in the decision-making process, the reasons for not discussing end-of-life dilemmas with the patient and family, whether a consensus was reached in the medical team about the decision, and whether advance directives existed. In addition, if a decision to limit therapy was taken, the physician was asked to note the life-support modalities withheld or withdrawn. The physicians of each ICU deposited the completed questionnaire in a sealed unmarked box. The several boxes collected from participating ICUs were mixed and opened all together at the end of the study.

For all patients, the following clinical and demographic data were extracted from the charts: age, gender, hospital and ICU length of stay, origin of admission (emergency department, medical ward, surgical ward, operating room, other ICU), admission diagnosis, chronic disorders (malignancy, acquired immunodeficiency syndrome (AIDS)/human immunodeficiency virus (HIV), cirrhosis, chronic heart failure of New York Heart Association (NYHA) classes III to IV, chronic respiratory insufficiency, chronic renal disease requiring dialysis, chronic neurologic or psychiatric disease), surgical status, Glasgow Coma Scale score (GCS) and Acute Physiology and Chronic Health Evaluation (APACHE) II scores on admission to the ICU, and APACHE II 24 hours before death.

Statistical analysis was performed to determine differences between the group of patients who received full support including unsuccessful CPR (group A), and the group of patients in whom therapy was limited in any way (including withholding of CPR, withholding of some form of life support besides CPR, and withdrawal of treatment (groups B, C, and D, consolidated)). Categoric variables were analyzed with the χ^2 ^test, and continuous variables with the *t *test. Differences were accepted as statistically significant when *P *< 0.05. All statistical tests were two-tailed.

The study protocol was approved by the Scientific Council and the Ethics Committee of Evangelismos Hospital, Athens, Greece. Informed consent was not required, because no interventions or treatments were given to the patients as part of this observational study, and the process of the study did not affect therapeutic decisions.

## Results

During the study, 2,040 patients (range, 66 to 763 patients per center) were admitted to the ICUs over a 9-month period (range, 3 to 20 months). Of the 2,040 patients, 464 (23%) died. Of the 464 patients, 132 were excluded, 48 because they were diagnosed with brain death, and 84 because they stayed in the ICU less than 48 hours. For 26 patients, information about the manner of dying was unavailable. Thus, 306 patients composed the study population. Their mean age was 64 ± 17 (SD) years, and their mean APACHE II score on admission to the ICU was 21 ± 7 (SD).

One hundred twenty-four (41%) patients received full support, including unsuccessful CPR. Limitation of life-sustaining therapy occurred in 182 (59%) patients: 148 (48%) died after withholding of CPR, 25 (8%), after withholding of other treatment modalities besides CPR, and nine (3%) after withdrawal of treatment.

Table 2 lists the demographic and clinical characteristics of patients according to whether therapy was limited. Patients in whom therapy was limited had a statistically significantly longer hospital and ICU stay, a lower admission GCS score, a higher APACHE II score 24 hours before death, and were more likely to be admitted with a neurologic diagnosis. Patients who received full support were more likely to be admitted with either a cardiovascular or a trauma diagnosis, and to be surgical rather than medical.

**Table 2:** Patient characteristics according to whether therapy was limited or not (n = 306)

Patient characteristics	No limitation (*n *= 124)	Any limitation (*n *= 182)	*P*
Age, years^a^	64 ± 17	65 ± 17	0.75
Male gender, *n *(%)	76 (61)	112 (62)	0.96
Hospital length of stay, days^b^	22 (9 to 36)	27 (13 to 44)	**0.01**
ICU length of stay, days^b^	10 (5 to 19)	15 (7 to 31)	**<0.01**
APACHE II on admission to the ICU^a^	20 ± 8	21 ± 7	0.22
GCS on admission to the ICU^b^	14 (8 to 15)	10 (6 to 15)	**<0.01**
APACHE II 24 hours before death^a^	23 ± 7	27 ± 7	**<0.01**
Having one or more chronic disorders,^c ^*n *(%)	64 (55)	114 (64)	0.12
Having malignancy, *n *(%)	29 (25)	48 (27)	0.72
Admission diagnosis, *n *(%)			
Cardiovascular	28 (23)	23 (13)	**0.02**
Respiratory	29 (24)	53 (29)	0.30
Gastrointestinal	21 (17)	31 (17)	0.97
Neurologic	9 (7)	40 (22)	**<0.01**
Sepsis	5 (4)	7 (4)	0.91
Trauma	17 (14)	13 (7)	**0.05**
Surgical, *n *(%)	68 (55)	79 (44)	**0.05**
Origin of admission, *n *(%)			
Emergency room	15 (13)	29 (16)	0.34
Medical ward	42 (35)	59 (33)	0.68
Surgical ward	37 (31)	53 (30)	0.78
Operating room	18 (15)	26 (14)	0.78
ICU of other hospital	7 (6)	12 (7)	0.78

^a^Mean ± SD. ^b^Median, quartiles. ^c^Malignancy, AIDS/HIV, cirrhosis, chronic heart failure NYHA III to IV, chronic respiratory insufficiency, chronic renal disease requiring dialysis, chronic neurologic or psychiatric disease. AIDS, acquired immunodeficiency syndrome; APACHE, Acute Physiology and Chronic Health Evaluation; GCS, Glascow Coma Scale; HIV, human immunodeficiency virus; ICU, intensive care unit; NYHA, New York Heart Association; SD, standard deviation.

The main factors influencing the physician's decision either to provide full support including CPR to patients of group A, or to use every available life-sustaining modality except CPR in patients of group B, were reversibility of illness and prognostic uncertainty; the physician's religious beliefs and legal concerns had minimal impact (Tables 3 and 4). Correspondingly, the most important factors affecting the decision either not to resuscitate patients of group B, or to withhold or withdraw life-sustaining treatment in patients of groups C and D, were unresponsiveness to treatment already offered, prognosis of underlying chronic disease, prognosis of acute illness, and future poor health; age was infrequently cited, whereas economic cost and lack of ICU beds played almost no role (Tables 5 and 6).

**Table 3:** Factors that influenced the decision to provide full support, including unsuccessful CPR, ranked by impact

Factor	Impact on the decision				
	
	No	Little	Moderate	Much	Ultimate
Reversibility of illness	8 (8)	6 (6)	14 (14)	16 (16)	54 (55)
Prognostic uncertainty	23 (23)	7 (7)	13 (13)	30 (31)	25 (26)
Age (years)	43 (44)	18 (18)	10 (10)	5 (5)	22 (22)
Relatives' opinion	45 (46)	13 (13)	12 (12)	8 (8)	20 (20)
Emotion/conscience	51 (52)	5 (5)	15 (15)	19 (19)	8 (8)
Physician's religious beliefs	65 (66)	6 (6)	10 (10)	15 (15)	2 (2)
Legal concerns	74 (76)	13 (13)	7 (7)	1 (1)	3 (3)
Patient's will	74 (76)	8 (8)	5 (5)	6 (6)	5 (5)
Bad communication with relatives	87 (89)	6 (6)	2 (2)	2 (2)	1 (1)
Family pressures	90 (92)	3 (3)	0 (0)	2 (2)	3 (3)
Disagreements within the medical team	90 (92)	5 (5)	0 (0)	0 (0)	3 (3)
Disagreements within the family	93 (95)	4 (4)	0 (0)	0 (0)	1 (1)

Data are presented as numbers (percentages) of patients. The respective section of the questionnaire was filled for 98 patients. CPR, cardiopulmonary resuscitation.

**Table 4:** Factors that influenced the decision to provide active support up to but not including CPR, ranked by impact

Factor	Impact on the decision				
	
	No	Little	Moderate	Much	Ultimate
Reversibility of illness	22 (16)	8 (6)	31 (22)	35 (25)	45 (32)
Prognostic uncertainty	46 (33)	16 (11)	33 (23)	29 (21)	17 (12)
Age (years)	81 (57)	18 (13)	13 (9)	11 (8)	18 (13)
Relatives' opinion	79 (56)	15 (11)	14 (10)	13 (9)	20 (14)
Emotion/conscience	85 (60)	12 (9)	16 (11)	16 (11)	12 (9)
Physician's religious beliefs	103 (73)	14 (10)	12 (9)	7 (5)	5 (4)
Legal concerns	118 (84)	13 (9)	4 (3)	4 (3)	2 (1)
Patient's will	125 (89)	8 (6)	4 (3)	3 (2)	1 (1)
Bad communication with relatives	129 (91)	7 (5)	2 (1)	1 (1)	2 (1)
Family pressures	125 (89)	9 (6)	4 (3)	2 (1)	1 (1)
Disagreements within the medical team	130 (92)	6 (4)	3 (2)	0 (0)	2 (1)
Disagreements within the family	134 (95)	4 (3)	1 (1)	1 (1)	1 (1)

Data are presented as numbers (percentages) of patients. The respective section of the questionnaire was filled for 141 patients. CPR, cardiopulmonary resuscitation.

**Table 5:** Factors that influenced the decision to withhold CPR, ranked by impact

Factor	Impact on the decision				
	
	No	Little	Moderate	Much	Ultimate
Unresponsiveness to treatment already offered	33 (23)	0 (0)	7 (5)	11 (8)	90 (64)
Prognosis of underlying chronic disease	17 (12)	3 (2)	6 (4)	29 (21)	86 (61)
Prognosis of acute illness	33 (23)	8 (6)	17 (12)	24 (17)	59 (42)
Future poor health	67 (48)	8 (6)	14 (10)	17 (12)	35 (25)
Preexisting poor health	74 (52)	12 (9)	11 (8)	17 (12)	27 (19)
Age (years)	96 (68)	14 (10)	7 (5)	6 (4)	18 (13)
Aggressiveness of treatment, discomfort disproportionate to expected benefit	95 (67)	19 (13)	5 (4)	10 (7)	12 (9)
Physical and psychological pain	81 (57)	17 (12)	14 (10)	16 (11)	13 (9)
Emotion/conscience	108 (77)	14 (10)	12 (9)	0 (0)	7 (5)
Relatives' opinion	120 (85)	9 (6)	6 (4)	2 (1)	4 (3)
Physician's religious beliefs	118 (84)	9 (6)	10 (7)	4 (3)	0 (0)
Economic cost	129 (91)	7 (5)	4 (3)	0 (0)	1 (1)
Patient's will	134 (95)	3 (2)	4 (3)	0 (0)	0 (0)
Lack of ICU beds	134 (95)	5 (4)	1 (1)	0 (0)	1 (1)

Data are presented as numbers (percentages) of patients. The respective section of the questionnaire was filled for 141 patients. CPR, cardiopulmonary resuscitation; ICU, intensive care unit.

**Table 6:** Factors that influenced the decision to withhold or withdraw treatment, ranked by impact

Factor	Impact on the decision				
	
	No	Little	Moderate	Much	Ultimate
Unresponsiveness to treatment already offered	2 (6)	0 (0)	3 (9)	4 (12)	24 (73)
Prognosis of underlying chronic disease	4 (12)	1 (3)	0 (0)	3 (9)	25 (76)
Prognosis of acute illness	2 (6)	1 (3)	3 (9)	7 (21)	20 (61)
Future poor health	8 (24)	0 (0)	1 (3)	6 (18)	18 (55)
Preexisting poor health	13 (39)	3 (9)	1 (3)	4 (12)	12 (36)
Aggressiveness of treatment, discomfort disproportionate to expected benefit	7 (21)	4 (12)	6 (18)	2 (6)	14 (42)
Physical and psychological pain	9 (27)	10 (30)	3 (9)	9 (27)	2 (6)
Age (years)	18 (55)	5 (15)	4 (12)	4 (12)	2 (6)
Emotion/conscience	19 (58)	7 (21)	3 (9)	3 (9)	1 (3)
Relatives' opinion	20 (61)	4 (12)	5 (15)	1 (3)	3 (9)
Physician's religious beliefs	25 (76)	3 (9)	3 (9)	2 (6)	0 (0)
Economic cost	31 (94)	1 (3)	1 (3)	0 (0)	0 (0)
Patient's will	32 (97)	0 (0)	0 (0)	0 (0)	1 (3)
Lack of ICU beds	33 (100)	0 (0)	0 (0)	0 (0)	0 (0)

Data are presented as numbers (percentages) of patients. The respective section of the questionnaire was filled for 33 patients. ICU, intensive care unit.

Only three (1%) patients were involved in end-of-life decisions; in two of these three cases, the patient expressed a request for limitation of life-sustaining treatment, which was ignored by the physician; in one case, the patient consented to receive full support (Table 7). Of the patients, 89% were mentally incompetent at the time of the decision; 5% were unaware of their diagnosis or prognosis or both; and 3% were judged to be unable to comprehend the dilemma posed. Advance directives were rare (1%).

**Table 7:** Participation of patient and relatives in the decision-making process by end-of-life category

	**A (*n *= 98)**^**a**^	**B (*n *= 140)**^**a**^	**C (*n *= 23)**^**a**^	**D (*n *= 8)**^**a**^	**Total (*n *= 269)**^**a**^
No patient or family involvement	68 (69)	129 (92)	10 (43)	5 (63)	212 (79)
Patient consented	1 (1)	0 (0)	0 (0)	0 (0)	1 (0.4)
Patient disagreed	1 (1)	0 (0)	0 (0)	0 (0)	1 (0.4)
Relatives consented	26 (27)	10 (7)	13 (57)	3 (37)	52 (19)
Relatives disagreed	0 (0)	1 (1)	0 (0)	0 (0)	1 (0.4)
Patient disagreed, but relatives consented	1 (1)	0 (0)	0 (0)	0 (0)	1 (0.4)
Relatives insisted despite physician' s recommendation to the contrary	1 (1)	0 (0)	0 (0)	0 (0)	1 (0.4)

Data are presented as numbers (percentages) of patients. ^**a**^Number of patients for whom the respective section of the questionnaire was filled.

**A**, full support including unsuccessful CPR; **B**, active support up to but not including CPR; **C**, withholding (not starting or escalating) some form of life support (besides CPR); **D**, withdrawal of existing treatment. CPR, cardiopulmonary resuscitation.

Relatives' participation in decision making occurred in 20% of cases and was more frequent when a decision to offer full support was made than when treatment was limited in any way (*P *< 0.01) (Table 7). Conversations were principally initiated by the physician (62%). Reasons for not discussing end-of-life practices with relatives were as follows: the family was thought not to understand (60%); the family was unavailable (25%); such a discussion was considered unnecessary by the physician (10%); or the family did not want to participate in the decisions (4%).

In 94% of cases, the medical team reached consensus about the end-of-life practice followed. Nurses were never included in consensus development, but were informed about the decisions. Almost always (98%), the attending physician stated that he or she was sure that he or she had made the right decision. Only 6% of patients in whom CPR was withheld had a written account of the "do not resuscitate" (DNR) decision present in their charts. However, decisions to forego (withhold or withdraw) life-sustaining therapy (besides CPR) were documented in the medical record in 52% of the corresponding cases.

The therapeutic interventions most frequently withheld/withdrawn were vasopressors/inotropes and dialysis. Other life-support modalities withheld/withdrawn are shown in Table 8. The median time from ICU admission to the decision to withhold treatment was 8.5 days (range, 0 to 129 days). The median time from withholding of therapy to death was 48 hours (range, 0.5 hours to 30 days). The median time from ICU admission to the decision to withdraw treatment was 14 days (range, 3 to 116 days). The median time from withdrawal of therapy to death was 32 hours (range, 1 hour to 4 days). The withholding or withdrawal decision was considered by physicians to have been timely in 79% of cases and inappropriately delayed in 21%.

**Table 8:** Life-support modalities withheld/withdrawn

Modality	Withholding (*n *= 25)	Withdrawal (*n *= 8)
Vasopressors/inotropes	19 (76)	5 (63)
Dialysis	10 (40)	2 (25)
Transfusions	5 (20)	-
Antibiotics	4 (16)	2 (25)
Mechanical ventilation	4 (16)	3 (37)
Parenteral nutrition	2 (13)	1 (13)

Data are given as numbers (percentages) of patients. Patients may have several life-support modalities withheld or withdrawn. The respective section of the questionnaire was filled for 33 patients.

## Discussion

The present multicenter study demonstrates that limitation of life-sustaining treatment is a common phenomenon in Greek ICUs; more than half of deaths are preceded by a decision to forego some form of supportive therapy. Nevertheless, in the vast majority of cases (>80%), the only limitation of treatment that takes place is withholding of CPR. Withholding of other life-support modalities besides CPR is not a routine practice, whereas withdrawal of treatment is quite infrequent. The observed rate of CPR use (40.5%) is consistent with data reported from southern countries (Greece, Israel, Italy, Portugal, Spain, and Turkey) in the European Ethicus study, and is much higher than the European mean (21%) [3]. In northern European countries, as well as in North America, the incidence of withholding and withdrawal of life-sustaining treatment reaches 90% of patients who die in the ICU [3,15].

A remarkable observation of the current study is that withdrawal of mechanical ventilation happens only on rare occasions. Although the same moral justification is required to withdraw one form of support or another [16], withdrawal of mechanical ventilation seems to be a taboo practice. Clearly, given that patients usually die soon after ventilator withdrawal, most Greek physicians see ventilator support as the ultimate tool in life support, which cannot be withdrawn without taking personal responsibility for the death of a patient.

International discrepancies in end-of-life practices have been considered to reflect cultural and religious differences [13,14,17]. However, our study indicated that religious faith did not exercise any noteworthy influence on physician attitudes. Perhaps religion affects physician attitudes in a less-obvious way, by being a part of the culture in which the physicians have grown up. Additional explanations that have been proposed for the lower frequency of limitation of treatment in southern countries comprise the ambiguous legal context, and the absence of guidelines from national scientific societies [1,10,18-20]. Still, we found that physician reluctance to withhold or withdraw treatment did not emanate from legal concerns. It seems that, in southern Europe as well as in the Middle and Far East, the traditional belief that life must be preserved at all costs is stronger than that in northern Europe and North America [11,19-21].

Despite the financial problems with which the Greek health-care system is confronted, economic cost was not proved to be a determinant of end-of-life decisions. Similarly, notwithstanding the scarcity of ICU beds, in almost no case was life support withheld or withdrawn on the basis of resource allocation.

In this study, the choice between providing full support and foregoing life-sustaining therapy was driven primarily by an evaluation of objective medical data, mainly the predicted reversibility of the underlying and acute conditions and the unresponsiveness to treatment already offered. Prognostic uncertainty contributed considerably to the decision not to withhold or withdraw life-preserving interventions, indicating physician perseverence until all hope of patient survival had vanished. When deciding to withhold or withdraw life-sustaining therapy (besides CPR), physicians seriously took into account the patient's preexisting and future poor health. Hence, physicians' perception of patients' quality of life seems to be a substantial factor in such decisions.

In contrast to previous research [3,5,6,8,9,12,22], we found no association between the limitation of treatment and the patient's age. Moreover, age was rarely cited as a factor prompting the decision to forego life support. This is an encouraging finding. It has been argued that old age alone is not a valid justification for refusing intensive care [23]. After all, the literature provides contradictory results as to whether the ICU mortality of elderly patients is significantly higher than that of young patients after adjustment for confounding factors [24-26].

Again, unlike in other studies [3,5,8,9,12,22,27], patients who received full treatment and those who underwent limitation of life-sustaining therapy did not differ in regard to the severity of illness on admission to the ICU (as measured by the APACHE II score) and the presence of comorbidities, including malignancy. Conversely, patients in whom treatment was withheld/withdrawn had a more protracted course, as reflected in their longer hospital and ICU stay, and a higher APACHE II score 24 hours before death. These findings imply that, for each patient, end-of-life practice was not determined by the initial clinical parameters, but it was gradually shaped on the basis of the disorder's unfavorable evolution, the development of an irreversible sequence of complications, and the progressive physiological deterioration.

Specific diagnostic categories (cardiovascular disease and trauma) were correlated with fewer limitation decisions. Furthermore, surgical patients were fully supported more often than were medical patients. On the contrary, patients admitted with a neurologic diagnosis were more likely to undergo limitation of treatment. These findings have two possible explanations. First, cardiovascular disease is deemed more reversible than is neurologic injury, which is viewed as a devastating irremediable damage. Second, in trauma as well as in many surgical patients, illness is sudden and unexpected, which may delay the recognition of futility and impede decision making.

We observed that death does not always ensue shortly after withholding or withdrawal of therapy; time from withholding of therapy to death may be as long as 1 month. This observation suggests the need for transfering patients whose death is not immediately imminent after limitation of treatment, to a suitable hospice, to administer appropriate palliative care.

Our data indicate that paternalism prevails in the Greek ICUs studied. The physician possesses a dominant role in the decision-making process and retains the final responsibility for end-of-life practice. Relatives' involvement in decision making is uncommon, and advance directives are rare. Respect for and confidence in medical authority are deep-rooted in Greek culture. Patients and families traditionally tend to entrust therapeutic decisions to physicians. In the same manner, end-of-life decisions are envisaged as purely clinical or professional judgments and are left to the doctor. Besides, most patients with chronic terminal illnesses do not have full knowledge of their diagnosis or prognosis. Nondisclosure is believed to protect patients from anxiety and depression, and to keep hope alive. Last, as has emerged from several studies, in southern European countries, the ethical principle of beneficence still overshadows autonomy [6,18,28-30].

The percentages of medical-record documentation of limitation decisions were low, a finding that confirms the results of the Ethicus study, which revealed a south-to-north difference regarding the presence of written accounts of such decisions [31]. Ideally, each patient's chart should have a complete documentation of the end-of-life practice. However, physicians may not believe this is necessary.

The strengths of the present study are the direct reporting of physicians' actions rather than theoretic responses to a survey's questionnaire, the prospective design, the enrollment of a sufficient number of consecutive patients from multiple centers, the anonymity, and the fact that data were collected not only about patients who died after limitation of life-support but also about patients who died despite ongoing active treatment. Exclusion of patients who died within 48 hours after admission to the ICU is a limitation of our study. We thought that, in this group of patients, dealing with end-of-life dilemmas is unusual, because, in most cases, important aspects of the previous medical history are unknown, and prognosis is uncertain.

Another limitation is that the validity of the questionnaire may be challenged, because it was not tested before the study. The questionnaire's structure was based on a literature survey of factors that influence end-of-life practice. Also, we did not evaluate the impact of patient race, ethnicity, religion, and socioeconomic status on end-of-life decisions. Yet, a large variation of these parameters does not exist in the Greek ICU population.

Finally, we did not investigate the possible association between physician characteristics (age, medical specialty, years of clinical experience) and his or her willingness to withhold or to withdraw life-sustaining therapies.

## Conclusions

This prospective multicenter study showed that limitation of life-sustaining treatment is a common phenomenon in the Greek ICUs studied. However, in a large majority of cases, it is equivalent to the withholding of CPR alone. Withholding of other therapies besides CPR is not routine, and withdrawal of support is infrequent. The main factor guiding the decision to limit therapy is unresponsiveness to treatment already offered. Economic cost and lack of ICU beds seem to play no role. As in other European countries, the paternalistic model predominates in decision making. By recording current medical practice and its motivations in end-of-life situations, our study helps to translate moral principles into legal and scientific guidelines. Such guidelines can use recent international recommendations as a baseline reference and adapt them to our local particularities.

## Key messages

• Limitation of life-sustaining treatment is a common phenomenon in the Greek ICUs studied. However, in most cases, it involves the withholding of CPR only.

• Withholding of other therapies besides CPR and withdrawal of support are infrequent.

• Unresponsiveness to treatment already offered is the main factor influencing the physician's decision to limit therapy.

• Medical paternalism prevails in the decision-making process.

• Death does not always ensue shortly after withholding or withdrawal of treatment; patients whose death is not immediately imminent should be transferred to suitable hospices.

## Abbreviations

AIDS: acquired immunodeficiency syndrome; APACHE: Acute Physiology and Chronic Health Evaluation; CPR: cardiopulmonary resuscitation; DNR: do not resuscitate; GCS: Glascow Coma Scale; HIV: human immunodeficiency virus; ICU: intensive care unit; NYHA: New York Heart Association; SD: standard deviation.

## Competing interests

The authors declare that they have no competing interests.

## Authors' contributions

GK contributed to the conception, design, and coordination of the study, the acquisition, analysis, and interpretation of data, and drafting the manuscript. VG and AT contributed to the conception and design of the study, acquisition, analysis, and interpretation of data, and revising the manuscript. ET contributed to the acquisition, analysis, and interpretation of data, and to revising the manuscript. P-MC-M contributed to the acquisition of data and revising the manuscript. IV, EM, VM, CR, AP, TK, GG, IF, and AK contributed to the acquisition of data. SN contributed to the conception, design, and coordination of the study, the acquisition, analysis, and interpretation of data, the general supervision of the research group, critically revising the manuscript for important intellectual content, and the final approval of the version to be published.
